# lncRNA involvement in hepatocellular carcinoma metastasis and prognosis

**DOI:** 10.17179/excli2018-1541

**Published:** 2018-09-04

**Authors:** Maryam Abbastabar, Mohammad Sarfi, Abolfazl Golestani, Ehsan Khalili

**Affiliations:** 1 Department of Clinical Biochemistry, Faculty of Medicine, Tehran University of Medical Sciences, Tehran, I.R. Iran

**Keywords:** hepatocellular carcinoma (HCC), long non-coding RNA (lncRNAs), metastasis, prognosis, liver

## Abstract

Eukaryotic lncRNAs are RNA molecules defined to be greater than 200 bp in length that are not translated to a protein and operate through several mechanisms, including participating in chromatin remodeling and methylation, influencing the integrity and stability of proteins and complexes, or acting as a sponge for miRNA inhibition. A number of recent studies have concentrated on the relationship between long non-coding RNAs (lncRNAs) and cancer. Hepatocellular carcinoma (HCC) is the most prevalent histological type of liver tumors, accounting for about 80 % of the cases worldwide. Lack of proper molecular markers for diagnosis of HCC and treatment evaluation is a significant problem. Dysregulated expression of HCC-related lncRNAs such as MEG-3, MALAT1, HULC, HOTAIR, and H19 have been identified and closely related with tumorigenesis, metastasis, prognosis and diagnosis. In this review, we summarized recent highlighted functions and molecular mechanisms of the most extensively studied lncRNAs in the pathophysiology of hepatocellular carcinoma and their potential for serving as probable therapeutic targets.

## Introduction

In humans, more than 85 % of the genome is transcribed. RNAs that do not encode proteins are called non-coding RNAs (ncRNAs). Non-coding RNAs are classified into two groups based on their size. One group is short RNAs with less than 200 nucleotides such as miRNA, siRNA, snoRNA and the other is long non-coding RNAs with more than 200 nucleotides. LncRNAs, in contrast with miRNAs, are less understood (Berretta and Morillon, 2009[4]; Haggar and Boushey, 2009[25]; ENCODE Project Consortium, 2012[17]; Guttman and Rinn, 2012[24]). XIST (X-inactive specific transcript) and H19 were the first lncRNAs discovered in the 1990s (Brannan et al., 1990[5]; Brockdorff et al., 1992[6]).

XIST has a role in inactivation of X-chromosome in female zygotes (Marahrens et al., 1998[68]). Many lncRNAs are transcribed and spliced by RNA polymerase II and several lncRNAs have both poly-A tail and 5´ cap (Derrien et al., 2012[13]). Similar to protein-coding genes, lncRNAs have certain epigenetic modifications like H3K4me3 in the promoter of the gene and H3K36me3 that are seen throughout their genome. They generally do not have functional open reading frames (ORFs). However, this discrepancy is obscured by the discovery of bifunctional RNAs that may possess both protein-coding and coding-free functions. Even though LncRNAs can be found in many tissues, the brain and central nervous system have the highest levels of expressed lncRNAs. The intracellular location of these molecules also varies, as they can be found in a wide range of intracellular components such as the nucleus, the cytoplasm, or in one or more focal regions of the cells. Their location may indicate their likely performance (Dinger et al., 2008[15]; Warden et al., 2008[101]; Ponjavic et al., 2009[79]).

The basis for nomenclature of lncRNAs is different. Some of them are named based on their role, such as PRAL (P53 Regulation-Association Long Non-Coding RNA), or based on their tissue expression such as HULC (Highly upregulated in liver cancer), while some are named based on their genomic location such as HOTAIR (Hox antisense intergenic RNA) (Warden et al., 2008[101]). LncRNAs have their own functional attributes due to their secondary structures; they usually have stem-loop secondary structures (Kino et al., 2010[44]). They interact with other biological molecules such as RNA, DNA, and protein, and other critical factors in promoting the physiological activity of natural cells. These molecules play important roles in biological processes through multiple mechanisms, such as acting as a sponge for miRNAs inhibition, participating in chromatin remodeling and also influencing the stability of proteins (Quinn and Chang, 2016[82]). Many of these molecules are associated with human diseases such as gastric cancer (Du et al., 2015[16]), breast cancer (Gupta et al., 2010[23]), colorectal cancer (Kogo et al., 2011[46]), and non-small-cell lung cancer (NSCLC) (Sun et al., 2014[84]). 

Hepatocellular carcinoma (HCC) is the most prevalent histological type of liver tumors, accounting for about 80 % of the cases (DeSantis et al., 2014[14]). This malignancy is the seventh most common carcinoma in men and the ninth in women (Okuda, 1992[75]). HCC has a wide variety of geographical disparity. It is more prevalent in China, Taiwan, Korea and other countries in Southeast Asia and Sub-Saharan Africa (Wang et al., 1991[100]). The main risk factors for hepatocellular carcinoma are age, sex, and cirrhosis, but the main cause is an overdose of alcohol or chronic infection with hepatitis B or hepatitis C viruses (Trevisani et al., 1996[88]; Velázquez et al., 2003[92]). Several signaling pathways have been shown to be critical players in HCC such as the Wnt/β Catenin (Herbst and Kolligs, 2007[30]), p53 (Hsu et al., 1993[32]), Ras (Liao et al., 1997[60]) and JAK/STAT pathway (Wormald and Hilton, 2004[102]). Although many diagnostic and treatment methods are available for HCC, including surgical resection, liver transplantation, radioembolization, radiation therapy, and molecularly targeted therapies (Attwa and El-Etreby, 2015[1]), the absence of suitable molecular markers for HCC diagnosis and therapy evaluation is a significant problem. Therefore, it is critical to develop novel strategies for early diagnosis, prognosis, prediction, and therapeutic targets of patients with HCC. In our study, we focused on abridging the conceivable functions and molecular mechanisms of the most extensively studied lncRNAs in HCC. Numerous lncRNAs have been reported to be involved in metastasis and prognosis of hepatocellular carcinoma. This review summarizes selected lncRNAs that are dysregulated in hepatocellular carcinoma studies, and discusses their mechanisms and clinical applications (Table 1(Tab. 1); References in Table 1: Cai and Cullen, 2007[7]; Chang et al., 2016[9]; Chapman et al., 2012[10]; Chen et al., 2013[11]; Coccia et al., 1992[12]; Geng et al., 2011[20]; Hashiguchi et al., 2013[26]; He et al., 2017[28]; Hou et al., 2017[31]; Huang et al., 2013[34]; Keniry et al., 2012[42]; Kinose et al., 2015[45]; Lay et al., 2000[48]; Li and Chen, 2013[51]; Li et al., 2014[58]; Li et al., 2016[54]; Li et al., 2016[55]; Li et al., 2017[50]; Liu et al., 2018[62]; Miyoshi et al., 2000[71]; Ni et al., 2017[73]; Ogasawara et al., 2010[74]; Panzitt et al., 2007[77]; Tu et al., 2014[91]; Vogelstein et al., 2000[93]; Wang et al., 2010[97]; Wang et al., 2014[95]; Wang et al., 2015[94]; Yan et al., 2016[107]; Yang et al., 2012[110]; Yang et al., 2018[112]; Yu et al., 2016[113]; Yuan et al., 2012[116]; Yuan et al., 2014[114]; Zhang et al., 2012[119]; Zhou et al., 2007[121]). 

## Metastasis-Associated Lung Adenocarcinoma Transcript 1 (MALAT-1)

MALAT-1, also known as nuclear enriched abundant transcript-2 (NEAT-2), is located at human chromosome 11q13.1 and mostly situated in nuclear speckles (Li and Chen, 2013[51]). This lncRNA interacts with serine/arginine (SR) proteins and regulates them and other splicing factors (Tripathi et al., 2010[89]). It is highly expressed in the brain (Bernard et al., 2010[3]) and overexpressed in many human carcinomas such as colorectal cancer (Yang et al., 2015[111]), non-small-cell lung cancer (Ji et al., 2003[37]), endometrial stromal sarcoma (ESS) (Yamada et al., 2006[106]), breast cancer (Guffanti et al., 2009[21]), and HCC. MALAT-1 has been elevated in both HCC lines and clinical tissue samples. Silencing of MALAT-1 by siRNA decreases cell proliferation and inhibits migration and invasion; therefore, it could be a novel biomarker for prediction of HCC recurrence following liver transplantation (Lai et al., 2012[47]). Hou et al. (2017[31]) demonstrated that hepatitis B virus X protein (HBx) could upregulate the long non-coding RNA MALAT-1 in HCC, and MALAT-1 could further influence the expression of latent transforming growth factor β-binding protein 3 (LTBP3), resulting in further progress and metastasis of HCC. In addition, Li et al. (2017[50]) reported that MALAT-1 acts as a molecular sponge for mir-146-5p to downregulate its expression in HCC. miR-146b-5p could obliterate amplification, migration, and invasion, and also induces apoptosis *in vitro *and* in vivo*. Remarkably, TNF receptor-associated factor 6 (TRAF6) has been justified as a lead target of miR-146b-5p in HCC and miR-146b-5p applies the cancer obliteration functions through repressing phosphorylation of Akt mediated by TRAF6. In addition, Liu et al. (2018[62]) suggested that MALAT-1 acts as a circular endogenous RNA for miR-195. Epidermal growth factor receptor (EGFR) is a direct target of miR-195. Sponging of miR-195 by MALAT1 exerts oncogenic effects since miR-195 is no longer able to suppress the downstream target EGFR.

## Maternally Expressed Gene-3 (MEG-3)

Maternally Expressed Gene 3 (MEG-3) is a human equivalent of mouse gene trap locus 2 (Gtl2) that is located at human chromosome 14q32.2 (Miyoshi et al., 2000[71]). Evidence suggests that MEG3 is expressed in normal tissues, whereas its expression is downregulated in tongue squamous cell carcinoma (Wang et al., 2014[98]), gastric cardiac adenocarcinoma (Guo et al., 2017[22]), nasopharyngeal carcinoma (Chak et al., 2017[8]) and gastric cancer (Peng et al., 2015[78]). This lncRNA is downregulated in HCC tissues, and its overexpression inhibits proliferation of the HCC Huh7 cell by negative modulation of miRNA-664 which reduces tumor growth, invasion, and metastasis in the orthotopic liver cancer model (Yang et al., 2012[110]; He et al., 2017[28]). This lncRNA also functions as a tumor inhibitor through P53-dependent and P53-independent pathways. Zhou and his colleagues (2007[121]) discovered that MEG3 induces P53 accumulation via repressing murine double minute 2 (MDM2) expression, which degrades p53. In addition it has been well documented that MEG3 has antitumor effects in the absence of p53 (Vogelstein et al., 2000[93]). Li et al. (2017[56]) found that miR-26a, as a tumor suppressor, and MEG3 decreased significantly in HCC compared to matched non-malignant tissues. MiR-26a binds to 3'-UTR of DNA methyltransferase3b (DNMT3B) and suppresses its expression, resulting in the upregulation of MEG3. In this way, miR-26a inhibits cell proliferation, migration, and invasion in HCC.

## Growth Arrest Specific 5 (GAS5)

GAS5, which accumulates in growth-arrested cells, is located at 1q25.1. This gene encodes multiple snoRNAs and lncRNAs that act as a ribo-repressor of the glucocorticoid and associated receptors (Coccia et al., 1992[12]). Various functions have been related to this transcript, including cell development prevention and apoptosis (Wang et al., 2018[99]). It has therefore been recognized as a potential tumor suppressor, with its inhibition linked to cancer in numerous diverse tissues. Tu et al. (2014[91]) revealed that the expression level of GAS5 is reduced in HCC compared to normal matched tissues. It also has been proven that GAS5 expression is associated with HCC tumor size, lymph node metastasis, and clinical stage. Chang et al. (2016[9]) reported that GAS5 participates in the epithelial mesenchymal transition (EMT) of HCC cells. Overexpression of GAS5 downregulates the vimentin and upregulates the E-cadherin level in hepatocellular carcinoma cells. There is a significant negative association between GAS5 and the vimentin level in vivo (Chang et al., 2016[9]). Vimentin is a 57 kDa, type III intermediate filament whose function is to maintain cell and tissue integrity. Vimentin is linked with tumor incursion and a poor prognosis in various types of cancers, including hepatocellular carcinoma (Hu et al., 2004[33]). These data suggest an essential role for GAS5 in the molecular etiology of HCC and implicate the potential application of GAS5 in HCC therapy.

## LncRNA with Associated Microvascular Invasion in HCC (MVIH)

MVIH is located at human chromosome 10q22 at RPS24 (Ribosomal Protein S24) gene which is overexpressed in HCC (Yuan et al., 2012[116]), and breast cancer (Lei et al., 2016[49]). Presently, little is known about MVIH. A current study showed that MVIH might control HCC cell vitality by sponging and repressing the expression of miR-199a (Shi et al., 2015[83]). Several reports have shown that miR-199a acts as a tumor repressor, promotes tumor cell apoptosis, and inhibits cell proliferation and migration in several cancers (Tian et al., 2014[87]; Wang et al., 2014[98]; Kinose et al., 2015[45]). Yuan et al. (2012[116]) found that MVIH could activate tumor-inducing angiogenesis by diminishing the secretion of phosphoglycerate kinase 1 (PGK1), which is a glycolytic enzyme that catalyzes the conversion of 1.3-diphosphoglycerate to 3-phosphoglycerate. This enzyme can be secreted by tumor cells and contributes to inhibition of angiogenesis (Lay et al., 2000[48]). The serum level of MVIH is inversely correlated with the level of PKG1. MVIH overexpression could predict the recurrence of early-stage HCC in patients (Yuan et al., 2012[116]). 

## Hox Antisense Intergenic RNA (HOTAIR)

This lncRNA's gene is situated inside the Homeobox C (HOXC) gene assemblage on chromosome 12 and is co-expressed with the HOXC genes (Wu et al., 2017[103]). HOTAIR recruits the polycomb repressive complex-2 (PRC2) and Lysine-Specific Histone Demethylase 1 (LSD1) to the specific site and regulates the HOXD gene expression (Yan et al., 2016[107]). This lncRNA has an essential function in the epigenetic control of gene expression. In this regard, HOTAIR is deregulated in various cancers like pancreatic cancer (Kim et al., 2013[43]), lung cancer (Loewen et al., 2014[64]), esophageal cancer (Lv et al., 2013[66]), and HCC. In HCC, HOTAIR is increased compared to non-cancerous tissues; it acts by activating the Wnt/β catenin signaling pathway and is associated with metastasis, differentiation, and early recurrence (Gao et al., 2016[19]). *In vitro* assays in the HCC cell line have demonstrated that knockdown of HOTAIR lncRNA diminishes cell proliferation and is associated with decreased levels of matrix metalloproteinase-9 and vascular endothelial growth factor protein, which are crucial for cell motility and metastasis (Geng et al., 2011[20]). On the other hand, HOTAIR promotes invasion and metastasis of HCC cells by enhancing EMT. This lncRNA acts as a miR-23b-3p sponge to positively regulate zinc finger E-box-binding homeobox 1(ZEB1), a transcription factor associated with EMT (Yang et al., 2018[112]). In addition, Wu et al. (2018[104]) reported that HOTAIR bestows its impacts on cell multiplication via controlling the opioid growth factor receptor expression, which is a negative biological regulator of cell proliferation in HCC.

## H19

H19 was the first imprinted non-coding transcript identified and has a highly conserved secondary structure (Cai and Cullen, 2007[7]). H19 is co-expressed with another maternally imprinted gene, insulin-like growth factor 2 (IGF-2) (Jones et al., 1998[39]). The H19 gene behaves as an oncogene and may serve as a potential new target for anti-tumor therapy (Matouk et al., 2007[70]). Increased expression of H19 RNA has been shown in a large group of tumors such as pancreatic cancer (Ma et al., 2014[67]), breast cancer (Zhang et al., 2016[118]) and HCC. Many studies have shown that H19 can interact with microRNAs and proteins. H19 acts as a sponge for miR-675 that is encoded in its first exon (Keniry et al., 2012[42]). Furthermore, H19 is associated with the protein complex hnRNP U/PCAF/ RNAPol II and activates the miR-200 family by increasing histone acetylation. Zhang et al. (2012[119]) demonstrated that H19 can alter the miR-200 pathway, thus contributing to mesenchymal-to-epithelial transition and repression of cancer metastasis.

## Highly Upregulated in Liver Cancer Non-Coding RNA (HULC)

HULC is located at human chromosome 6p24.3 and is the first ncRNA with highly specific upregulation in HCC. It can be detected in the blood of HCC patients (Panzitt et al., 2007[77]). This lncRNA may act as an endogenous sponge that downregulates a series of miRNAs' activities, including miR-372 and miR-200a-3p. Suppression of miR-372 reduces the translational repression of PRKACB (cAMP-dependent protein kinase catalytic subunit beta), which then triggers phosphorylation of CREB. Binding of phospho-CREB to the HULC promoter activates HULC expression (Wang et al., 2010[97]). HULC expression is not confined to HCC alone, but also to those colorectal carcinomas that metastasize to the liver (Matouk et al., 2009[69]). Li et al. (2016[54]) suggested that miR-200a-3p is negatively regulated by HULC, and HULC functions as a ceRNA to mediate EMT via up-regulating ZEB1 in HCC cells. Xiong and his colleagues (2017[105]) found that ectopic expression of HULC stimulates the autophagy of HCC cells via harmonizing silent information regulator 1 (Sirt1) protein and dampening of HULC sensitized HCC cells to antitumor reagents through suppressing protective autophagy. In addition, it has also been found that HULC and Linc00152 can act as novel biomarkers in predicting the diagnosis of HCC, and a combination of HULC, Linc00152, and AFP has the highest prediction value in HCC (Li et al., 2015[52]).

## Dresh

Hepatitis B virus (HBV) has an important role in human hepatocellular carcinoma (HCC). Many non-coding RNAs including miRNAs such as miR-18a (Liu et al., 2016[63]), mir-148a (Yuan et al., 2012[115]), mir-21 (Qiu et al., 2013[81]) and lncRNAs regulated by HBx in HCC have important biological functions in cell proliferation, apoptosis, invasion, and metastasis. HBx protein can decrease lncRNAs whose expression is downregulated by HBx (termed lncRNA-Dreh). LncRNA-Dreh acts as a cancer inhibitor and could attach to the intermediate filament protein vimentin, suppresses its expression, and alters the cytoskeletal structure and prevents tumor metastasis (Huang et al., 2013[34]).

## MDIG

MDIG was initially discovered as a mineral dust-induced transcript from coal miners' alveolar macrophages (Zhang et al., 2005[120]). The expression of MDIG is controlled by the c-Myc oncogene and termed as myc-induced nuclear antigen 53 (Mina53) (Ogasawara et al., 2010[74]). High levels of MDIG expression have been found in lung cancer (Lu et al., 2009[65]), renal cell carcinoma (Ishizaki et al., 2007[36]), lymphoma (Teye et al., 2007[86]), neuroblastoma (Fukahori et al., 2007[18]), esophageal squamous cell carcinoma, and HCC (Tsuneoka et al., 2004[90]). Chen et al. (2013[11]) demonstrated that MDIG participates in modification of H3K9me3 to impact the heterochromatin structure of the genome, and the expression of genes important for cell proliferation or transformation. Ogasawara et al. (2010[74]) proposed that Mina53 expression is accelerated in HCC with a lower histological grade, larger diameter, or cell growth competence, and Mina53 is linked to biological malignancy of HCC.

## ZEB1-AS1

Zinc finger E-box-binding homeobox 1 is a protein encoded by the ZEB1 gene in humans. There is a non-coding antisense transcript emanating from the promoters of ZEB1, ZEB1 antisense1 (ZEB1-AS1). ZEB1-AS1 is frequently upregulated in HCC samples, especially in metastatic tumor tissues, and may serve as a valuable prognostic biomarker for HCC (Li et al., 2016[55]). Hashiguchi et al. (2013[26]) revealed that positive ZEB-1 expression and loss of E-cadherin expression correlate with a poor prognosis and progression of HCC through their effect on the progression of EMT.

## UC.134 lncRNA

UC.134 is a novel lncRNA that inhibits liver tumor development by suppressing the CUL4A-mediated ubiquitination of LATS1 and augmenting YAP phosphorylation. Overexpression of uc.134 suppresses HCC cell proliferation, invasion, and metastasis *in vitro *and* in vivo*. The use of this lncRNA may provide a favorable therapeutic method by repressing YAP and stimulating Hippo kinase signaling (Ni et al., 2017[73]).

## PVT-1 and UC200mbe.2

The lncRNA plasmacytoma variant translocation 1 (PVT1) is 1716 nucleotides long. It is located at 8q24.21 and is a recently identified long non-coding RNA. The human PVT1 oncogene level has been discovered to be high in a succession of human cancers (Barsotti et al., 2012[2]; Chapman et al., 2012[10]). Yu et al. reported that lncRNAs PVT1 and uc002mbe.2 are upregulated in the sera of HCC patients compared to healthy controls (Yu et al., 2016[113]). They demonstrated that amalgamation of 2 lncRNAs in the serum produces a new supplementary approach for HCC diagnosis. In addition, Wang et al. (2014[95]) found that oncofetal long non-coding RNA PVT1 stimulates multiplication and acquisition of stem cell-like properties in hepatocellular carcinoma cells through stabilizing the NOP2 protein. The NOP2 nucleolar protein can intensify nucleolar activities and stimulate cell proliferation by modifying the cell cycle and the hPVT1/NOP2 pathway. It is also involved in promoting carcinogenesis and cell proliferation and acquiring stem cell-like properties in HCC cells.

## LncRNA-UCA1 and WRAP53

Urothelial carcinoma associated antigen 1 (UCA1) is a bladder cancer-specific lncRNA with a total length of 1439 bp that is located at 19p13.12 (Li et al., 2014[59]). UCA1 is a common molecular marker for lymph node metastasis and prognosis in various cancers, including colorectal cancer, breast cancer, esophageal cancer, lung cancer, and pancreatic cancer (Ni et al., 2015[72]; Wang et al., 2015[96]; He et al., 2017[27]). The expression of serum UCA1 is significantly higher in patients with HCC, allowing differentiation of HCC from benign liver disease and healthy controls. High expression of serum UCA1 is significantly associated with a high tumor grade, large tumor size, positive vascular invasion, and advanced TNM stage (Heng et al., 2018[29]). Kamel et al. (2016[40]) provided evidence that lncRNA-UCA1 and WD repeat containing antisense to TP53 (WRAP53) are highly expressed in the serum of HCC patients and HCV patients. They reported that lncRNA-WRAP53 expression is a useful prognostic marker for RFS in HCC. Wang et al. (2015[94]) demonstrated that UCA1 may act as an endogenous sponge by directly binding to miR-216b, and downregulation of miR-216b expression results in repression of fibroblast growth factor receptor1 (FGFR1) expression and activation of an FGFR1/ERK signaling pathway in HCC. Therefore, upregulated lncRNA-UCA1 levels in HCC tissues are associated with the TNM stage, metastasis, and postoperative survival.

## ATB

LncRNA activated by transforming growth factor beta (TGF-β) (lncRNA-ATB) is involved in cell proliferation and metastasis in a variety of cancers, including renal cell carcinoma, colorectal cancer, non-small-cell lung cancer, glioma, etc. (Iguchi et al., 2015[35]; Qiu et al., 2017[80]; Ke et al., 2017[41]; Li et al., 2017[59]). In addition, this lncRNA is upregulated in hepatocellular carcinoma metastases and is associated with a poor prognosis. LncRNA-ATB upregulates ZEB1 and ZEB2 by competitively binding to the miR-200 family, thereby inducing EMT and invasion (Yuan et al., 2014[114]). Li et al. (2018[57]) demonstrated that Astragaloside IV (AS-IV) significantly downregulates lncRNA-ATB expression in a dose- and time-dependent manner in HCC cells. AS-IV represses EMT and migration of HCC cells. Likewise, through downregulation of lncRNA-ATB, AS-IV inactivates IL-11/STAT3 signaling, induces HCC cell apoptosis, and reduces HCC cell viability.

## Other lncRNAs in HCC

Recent studies have established that many other lncRNAs are abnormally regulated in HCC, including lncRNA-Low Expression in Tumor (lncRNA-LET), located at 15q24.1. It is involved in cancer suppression in numerous tumor types, such as cervical cancer (Jiang et al., 2015[38]), nasopharyngeal carcinoma (Sun et al., 2015[85]), lung adenocarcinoma (Liu et al., 2016[61]), and HCC. Hypoxia can suppress lncRNA-LET by decreasing the histone H3 and H4 acetylation levels in its promoter region. Furthermore, downregulation of lncRNA-LET may affect the accumulation and stability of HIF-1a mRNA under hypoxic conditions. It is worth noting that the transcript levels of the endogenous hypoxia marker CA9 are inversely correlated with the levels of lncRNA-LET in primary HCC tissues, and downregulated expression of lncRNA-LET is associated with HCC metastasis (Olive et al., 2001[76]; Yang et al., 2013[108]). The High Expression in HCC lncRNA (termed lncRNA-HEIH) is an oncogenic lncRNA with a high expression in hepatocellular carcinoma. This lncRNA facilitates tumor growth through enhancing Zeste Homolog 2 in humans and may act as a critical regulatory factor in HCC progression (Yang et al., 2011[109]). P73 antisense RNA 1 T (TP73-AS1) is located at 1p36 on the human chromosome and is associated with cell proliferation and tumor progression (Zang et al., 2016[118]). Previous studies have shown that TP73-AS1 might be upregulated in HCC. TP73-AS1 modulates HCC cell proliferation by miR-200a-dependent HMGB1/RAGE regulation. TP73-AS1 might compete with HMGB1 for miR-200a binding to inhibit miR-200a expression. High lncRNA-TP73-AS1 expression in HCC is associated with a poorer prognosis. This lncRNA might play a key role in regulating the proliferation of HCC cells and may be a potential therapeutic target for HCC treatment (Li et al., 2017[53]).

## Conclusions

Hepatocellular carcinoma is one the most prevalent and aggressive human malignancies. Despite the use of diverse treatment methods, clinical prognosis remains poor. A number of recent studies have focused on the functions of lncRNAs in the initiation and progression of HCC. Nonetheless, our present understanding of lncRNAs is limited. Further investigations are essential to clarify the biological functions and molecular characteristics of lncRNAs in HCC.

## Acknowledgements

This work was financially supported by grants (95-01-30-31634) from the Deputy of Research, Tehran University of Medical Sciences.

## Conflict of interest

The authors declare that they have no conflict of interest.

## Figures and Tables

**Table 1:**
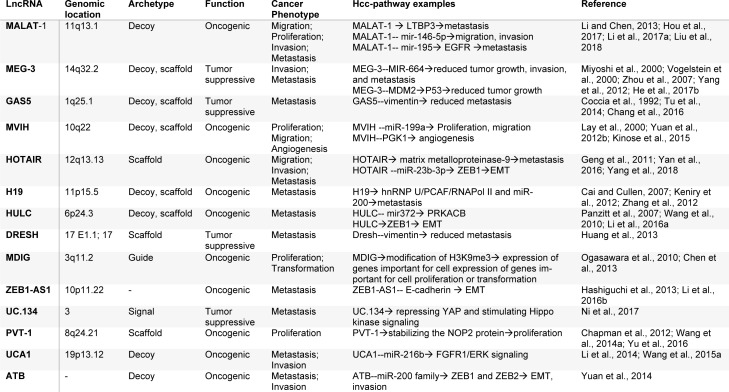
Summary of lncRNAs involved in the hepatocellular carcinoma
